# District health managers’ perceptions of supervision in Malawi and Tanzania

**DOI:** 10.1186/1478-4491-11-43

**Published:** 2013-09-05

**Authors:** Susan Bradley, Francis Kamwendo, Honorati Masanja, Helen de Pinho, Rachel Waxman, Camille Boostrom, Eilish McAuliffe

**Affiliations:** 1 Centre for Global Health, University of Dublin, Trinity College, Dublin, Ireland; 2 University of Malawi, College of Medicine, Centre for Reproductive Health, Blantyre, Malawi; 3 Ifakara Health Institute, Dar Es Salaam, Tanzania; 4 Averting Maternal Death and Disability Program (AMDD), Heilbrunn Department of Population and Family Health, Mailman School of Public Health, Columbia University, New York, NY, USA

**Keywords:** Supervision, Mid-level cadres, Malawi, Tanzania, District health management, Supervision paradigm, Measuring performance

## Abstract

**Background:**

Mid-level cadres are being used to address human resource shortages in many African contexts, but insufficient and ineffective human resource management is compromising their performance. Supervision plays a key role in performance and motivation, but is frequently characterised by periodic inspection and control, rather than support and feedback to improve performance. This paper explores the perceptions of district health management teams in Tanzania and Malawi on their role as supervisors and on the challenges to effective supervision at the district level.

**Methods:**

This qualitative study took place as part of a broader project, “Health Systems Strengthening for Equity: The Power and Potential of Mid-Level Providers”. Semi-structured interviews were conducted with 20 district health management team personnel in Malawi and 37 council health team members in Tanzania. The interviews covered a range of human resource management issues, including supervision and performance assessment, staff job descriptions and roles, motivation and working conditions.

**Results:**

Participants displayed varying attitudes to the nature and purpose of the supervision process. Much of the discourse in Malawi centred on inspection and control, while interviewees in Tanzania were more likely to articulate a paradigm characterised by support and improvement. In both countries, facility level performance metrics dominated. The lack of competency-based indicators or clear standards to assess individual health worker performance were considered problematic. Shortages of staff, at both district and facility level, were described as a major impediment to carrying out regular supervisory visits. Other challenges included conflicting and multiple responsibilities of district health team staff and financial constraints.

**Conclusion:**

Supervision is a central component of effective human resource management. Policy level attention is crucial to ensure a systematic, structured process that is based on common understandings of the role and purpose of supervision. This is particularly important in a context where the majority of staff are mid-level cadres for whom regulation and guidelines may not be as formalised or well-developed as for traditional cadres, such as registered nurses and medical doctors. Supervision needs to be adequately resourced and supported in order to improve performance and retention at the district level.

## Background

In many African countries, such as Tanzania and Malawi, mid-level cadres are a core component of the health system. However, insufficient and ineffective human resource management (HRM) of these staff constrains their ability to provide patients with high quality care [1-7]. Effective management of human resources requires that workers know exactly what tasks they are expected to perform, have the necessary skills and resources to perform these tasks, and receive feedback that assists them in improving their performance [8]. Supervision is central to this - it is thought to play an important role in the performance and motivation of health workers [9-12] and is particularly relevant in the context of task shifting [13-15]. While it is difficult to be certain of the long-term effectiveness of supervision activities in low-income contexts due to limited published evidence, supervision from higher to lower levels of the health service is widely recommended as a mechanism for supporting staff and ensuring quality of care [16].

‘Supervision’ is an ill-defined, complex activity [17]. In many resource-constrained settings it has its roots in hierarchical notions of the supervisor as the overseer [18], whose role is ensuring that the health system’s requirements are met, rather than addressing the development of skills and competencies of individual health workers [19]. In this context supervisory visits are the responsibility of external supervisors from the District Health Management Team (DHMT), and the supervision paradigm is commonly one of periodic inspection and control, rather than support. There is broad consensus that this is not effective [19,20] and that a widespread lack of recognition or reward for good performance leaves health workers with little incentive to perform well [21]. Recognition is a vital aspect of supervision that is all too often neglected. It plays a key role in the motivation and retention of health workers [22,23].

There is growing impetus for a move towards supportive supervision, which is defined as “an approach to supervision that emphasizes joint problem-solving, mentoring and two-way communication between the supervisor and those being supervised” [19]. This form of supervision promotes quality at all levels of the health system by strengthening relationships, optimizing the allocation of resources and fostering high standards and teamwork. Evidence of a conceptual move from traditional to supportive supervision exists in policy documents in many low-income countries, but is less apparent in practice changes at the district level [17]. This is compounded by a lack of clarity on the core elements of supervision as well as continuing debate, particularly in the nursing literature, on the boundaries between ‘clinical’ and ‘managerial’ aspects of supervision [24-26]. There is broad agreement in the health professions that supervision has three functions – management, education and support [27] – but less consensus on whether the same person should carry out these roles [24,28]. However, managerial supervision and support are seen as the foundation that is necessary to allow clinical supervision to function [26].

Tanzania and Malawi, the two countries involved in this study, have both increased their commitments to addressing their human resources for health constraints. Malawi has been engaged in a comprehensive national scale-up of health care workers. An ambitious Emergency Human Resources Programme (EHRP) was incorporated into the 2004 Health Sector Wide Approach as one pillar of a plan to deliver an Essential Health Package [29]. An integrated supervision checklist was developed to facilitate regular monitoring and supervision of service delivery at the operational levels [30]. The checklist was designed as a guide for use by zonal officers in their supervision of DHMTs and by the DHMT to supervise the facility staff in the districts for which they were responsible. There is also programme-specific supervision for key areas, such as HIV/AIDS and Integrated Management of Childhood Illness.

Malawi’s Ministry of Health has also committed itself to accelerating the reduction of maternal and neonatal death [31]. To achieve this goal, the government has expanded the number of cadres who are trained and authorised to perform the emergency obstetric care (EmOC) signal functions^a^, with delegation of some of these tasks to registered nurse-midwives, nurse-midwife technicians, clinical officers and medical assistants. This has clear implications for the need for effective, supportive supervision.

Tanzania has its own commitments to address human resource constraints [32,33] and reduce maternal mortality by scaling up provision of emergency obstetric care [34]. Health sector strategic plans now target urgent performance management and productivity issues by focusing on improved supervisory support and employee relations [32,33]. New supportive supervision guidelines [35] emphasise a process of problem solving, communication, teamwork and quality improvement, but there are still challenges and shortcomings to effective integration and implementation [36].

Responsibility for the management of health care services has been decentralised in Tanzania. At the regional level there is supervision and support of Council Health Management Teams (CHMTs). The CHMTs are responsible for implementation and evaluation at the district level. CHMT staff use a number of tools to monitor health programmes and services. The MTUHA (Health Management Information System) hospital data book has been in use since 1994 and is submitted to the CHMT every quarter. It provides a record of facility level indicators, logs all supervisory visits, and contains a table for problems identified and suggested solutions. More recently an Open Performance Review and Appraisal System (OPRAS) for the public service was introduced, to provide an open, formalised system for benchmarking and assessing staff performance [32]. At the time of data collection (October to December 2008) OPRAS was still being rolled out across the health sector, so its impact had not yet been documented.

The Health Systems Strengthening for Equity (HSSE): The Power and Potential of Mid-Level Providers project aimed to support health system strengthening for equity in Africa by building an evidence base on the role of mid-level cadres in maternal and neonatal health. HSSE was a large, mixed methods study that took place in Malawi, Tanzania and Mozambique. All quantitative and qualitative data were collected concurrently. This paper reports on the qualitative findings from that study in Malawi and Tanzania only. Analysis of the quantitative element of this research [37] provided robust evidence of the impact of supervision on health worker outcomes such as job satisfaction and intention to leave. It also identified differences in the types and frequency of supervision reported in Malawi and Tanzania. This evidence supports the need for systematic supportive supervision. Given that district personnel are responsible for carrying out supervision, it is important to examine their understanding of the role and purpose of this crucial aspect of the HRM system. It is also necessary to identify how the paradigm in which they operate and the challenges they face impact on regular supportive supervision of staff in primary health care facilities. The qualitative component of the HSSE research explored, *inter alia*, the perceptions of C/DHMT members on supervision practices in their respective districts and is reported here.

## Methods

This exploratory qualitative study took place as part of the larger HSSE project. Semi-structured, in-depth individual interviews were conducted with C/DHMT personnel in Malawi and Tanzania. The interview guide was based on *a priori* themes arising from the literature and was designed to elicit the perceptions of these personnel on a range of human resource issues. A comprehensive set of over 40 open-ended questions and additional relevant prompts was developed. These addressed seven key thematic areas related to human resource management, but maintained sufficient flexibility to allow for emerging themes to be evoked. The key areas of interest were: the autonomy of the district team; the current human resources situation; job descriptions and roles; supervision and performance assessment; working conditions, workloads and the work environment; motivation; and education and training.

### Sample

The data for this component of the HSSE research were gathered from a purposive sample of C/DHMT personnel in a subset of the districts selected for the main project. The qualitative researchers were part of the full HSSE data collection team and travelled with them through all the districts that were included in the HSSE project’s sampling frame. They were asked to interview C/DHMT personnel in two districts in each of five geographical zones in Malawi, and in two districts in each of eight selected regions in Tanzania. This sample size was deemed sufficient to provide a comprehensive overview of the perceptions of these cadres on human resource issues. In Malawi the key cadres targeted were District Health Officer (DHO), District Nursing Officer (DNO), or a Human Resources Officer in districts where this cadre was available. For Tanzania the key CHMT personnel were District Health Secretary (DHS), Reproductive and Child Health (RCH) Coordinator and District Medical Officer (DMO).

In both countries the research teams were directed to obtain interviews with all three key personnel, but C/DHMTs were extremely busy and this was not always possible. The researchers started trying to obtain interviews in the first district they visited in each region by making appointments with relevant senior staff. If they were unable to secure at least two interviews in that district they waited until the project team reached the next district, then tried again. This process continued until they had secured the required quota of interviews. Data were only included from districts where at least two of the key members of the C/DHMT were available to be interviewed at the time of data collection. Only two single interviews in Malawi had to be excluded from the analysis.

In both countries the teams met or exceeded their data collection target. In Malawi, 20 interviews were carried out in 10 of the 24 eligible districts. In Tanzania there were 47 eligible districts and a total of 37 interviews were conducted in 16 of these districts.

### Data collection

Data collection took place from October to December 2008. The Tanzanian research team consisted of eight experienced researchers who were either employees of Ifakara Health Institute or identified from Ifakara Health Institute’s database of researchers. Most were educated to Bachelor degree level. The Malawian team included three experienced researchers who were educated to at least Bachelor degree level, and there were two clinical officers. A one-week training programme on the HSSE project and methods was conducted with all research team members in each country prior to commencing data collection.

Interviews were conducted in English in Malawi and in Kiswahili in Tanzania. All interviews lasted approximately 1 to 1.5 hours. The objectives of the study were explained and confidentiality was assured. All data and records were rendered anonymous through the use of a unique identity number. Informed, signed consent was obtained from every respondent and all interviews were tape-recorded. Interviews were transcribed verbatim using Microsoft Word (Microsoft, Redmond, WA, USA). The Kiswahili transcripts were then translated into English by researchers fluent in both languages.

### Data analysis

All Word files were exported to NVivo8 software (QSR International Pty Ltd, Doncaster, Victoria, Australia) for thematic analysis. The analysis team consisted of two experienced researchers, one in Malawi and one in Tanzania (who did the coding), an experienced qualitative researcher (SB), and one of the study Principal Investigators (EM) who performed random checks on the coding. Emerging themes were developed through inductive and deductive processes [38]. The initial analysis used a coding framework, based on the thematic areas covered by the research questions, to generate top-level categories (tree nodes). The design of the interview schedule allowed the data to be auto-coded into these tree nodes. A detailed description of the expected content of each tree node was used by the analysis team to validate the content of each one, ensuring that all data within a node were true to the description of that node. Data that were relevant to other top-level nodes were also cross-coded into these nodes. The next phase of analysis involved bottom-up coding, with the team identifying and agreeing key subcategories emerging from each tree node. These data were coded into additional sub-codes (child nodes). The analysis team discussed their coding and interpretation of the transcripts in detail in order to improve inter-coder reliability.

One main area of the analysis explored responses to the interview questions about supervision and performance. The emergence of the central role of supervision in job satisfaction and retention as a key finding in the quantitative data warranted a deeper focus on the supervision-related aspects of the qualitative data. References to supervision permeated other sections of the data, so the coding exercise was further refined to gain a more nuanced and textured understanding of C/DHMT perceptions of this salient factor. The research team clustered related codes under broader categories to interpret the data and then used a process of synthesis to draw out five key themes.

The study was approved by the Global Health Ethics Committee, Trinity College, Dublin, and by the Institutional Review Boards of Columbia University, New York, the College of Medicine, Malawi, and Ifakara Health Institute, Tanzania.

## Results

Five major thematic areas emerged: the current supervision paradigm; why supervision is important; supervision in practice; assessing performance; and challenges to implementation.

### The current supervision paradigm

#### Malawi

The picture emerging from the interviews in Malawi was of a supervision paradigm focused on periodic inspection and control. Much of the language was couched in terms of fault-finding, poor performance and weakness. Respondents spoke of health workers being “corrected on their shortfalls” and wanted feedback so “we would know the weaknesses of that person” or “congratulate what they did well and rebuke them on what they did not do”. There were fewer references to supervision in terms of its potential to support staff, mentor them or recognise achievement. However, there were voices recognising the need for a change to a different form of supervision. These respondents wanted supervision that was more supportive of health workers, articulating a desire for a system that helped health workers address the challenges they face and acknowledged the good work that they do. They also spoke of the need to move from supervision as a periodically occurring activity to an ongoing, continuous process.

#### Tanzania

The paradigm expressed by CHMT members had an emphasis on assisting and supporting health workers. Many respondents talked explicitly of practicing ‘supportive supervision’.

“We do supportive supervision in health facilities. It means observing strengths and weaknesses, listening to the employees themselves as they give their views on the services they provide. After supervision they give feedback as to what was seen there, what needs to be improved. They apply what would need to be added in order to provide better health services.” (RCH Co-ordinator, 482)

Language such as “improve”, “instruct”, “advise”, “congratulate”, “assist”, “together” and “listen” was common and there seemed to be a focus on improvement, teaching and problem solving. “We should strategise for improvement. We sit, we talk, we discuss, at least trying to improve the quality.” (RCH Co-ordinator, 253) CHMT members were enthusiastic about the benefits of supportive supervision for both health workers and supervisors but recognised that some staff still did not value supervision and saw the supervisory team as coming to assess and judge them.

### Why supervision is important

#### Retention, motivation and performance

##### Malawi

There was a growing recognition among Malawian respondents of the importance of supervision to retention. “I always believe in supportive supervision. If you supervise these people regularly the chances of you retaining them are very high, unlike when you are not supervising them.” (DNO, 262) Another respondent felt it was important for management to see how difficult conditions were for nurses where staff shortages left them struggling to cover labour, antenatal and postnatal wards. He thought it was valuable for staff to be visited and felt that this could support them in addressing challenges. “When you go to do the supervision you see that really they are tired and frustrated.” (Deputy DHO, 252)

##### Tanzania

Managers in Tanzania displayed a robust appreciation of the importance of supervision and were positive about the structures in place to support staff. They felt it was a constructive way to improve motivation and performance in facilities, as it made staff feel appreciated. “…if you go there regularly they feel good and their performance improves.” (RCH Co-ordinator, 253) Supportive supervision was seen as a way to develop good management–staff relations and to demonstrate that their work was valued by the district. “…you should value your staff, I mean respecting one another…if you are capable you can motivate them so that they can see that you value their work.” (RCH Co-ordinator, 362) Two-way communication was appreciated as a critical factor in staff motivation. It was also described as an important mechanism to create team spirit by ensuring that workers were able to express their opinions and make suggestions to management, and allowing managers to ensure that lower cadres received information and support. “For the providers to have good work morale, the first thing is to have meetings where they can speak about their concerns and these can be dealt with.” (RCH Co-ordinator, 141)

#### Quality of care

##### Malawi

DHMT respondents appreciated that maternity differed from other departments because of overwhelming workloads and staff working in emergency mode for much of the time. “…they should…work hand in hand or close relationship with someone who is more senior to them…rather than just being left alone and hoping that they will manage all these things by themselves.” (Acting DHO, 311) The need for effective supervision or mentoring for cadres providing emergency obstetric care was clear to many respondents, but this had become more of a concern with the influx of large numbers of newly qualified staff due to the pre-service training element of the EHRP.

“…large numbers is nothing on its own. It is better to have numbers of good quality. So, they may produce [new staff] but they need to be followed up, supervised and possibly mentored properly when they start working. Not that after the training just dump them…make sure that when they recruit staff....they are monitored properly and again they are supervised, they are supported, to make sure that they meet the standards. Um, that is something to me that is very important step that we need to be taking.” (DHO, 162)

In addition, respondents thought that some of the new health workers did not always have the confidence or practical experience to perform the functions for which they had, theoretically, been trained. “…we don’t have the cadre that qualify right away from the college to do emergency obstetric care. They have to be trained, on job training…they need to be further reoriented to handle the basic emergency obstetric care…” (DHO, 121) In some districts where this was an issue, or where health worker cadres with EmOC skills were in short supply, this training was seen as part of the supervision process. “…it’s like on job training because of now we have a full time safe motherhood supervisor who goes out in the health centres one full day at the particular health centre…to teach them on EmOC issues and just to make supportive supervision.” (Deputy DHO, 252)

##### Tanzania

In Tanzania supportive supervision was seen as a way of disseminating new ideas and techniques and informing staff of changes in policy and guidelines. However, there were contradictions between respondents regarding how well maternity staff were supervised. CHMT respondents in some districts felt “…in reproductive issues we were very close to them and their work was better…” (RCH Co-ordinator, 253) Others were concerned that “…the way we are doing supervision to health workers who are providing emergency services during delivery it is not good to be honest. We don’t have that close supervision to tell them that you are supposed to do 1,2,3…sometimes people are doing things based on experience.” (DHS, 441) Additional difficulties arose when staff exceeded their scope of practice in emergency situations or due to staff shortage. “…the health providers they have deviated so much, these medical attendants he/she attend a patient, he/she gives injection, medicine, and sometimes performs delivery services, at the same time he/she has responsibility of doing cleanliness…” (Assistant DMO, 363)

### Supervision in practice

#### Malawi

The DHMT aimed to visit all health facilities on a quarterly basis, with one respondent characterising this as “regular management supervision”. Multi-disciplinary teams carried out integrated supervisory visits to assess all aspects of service level performance, while specific teams (such as the Safe Motherhood and RCH Co-ordinators for maternal health) supervised specialities and had the flexibility to visit facilities more frequently. There was little mention of a system or clear process, other than the use of a checklist. Staff who were based in the district hospitals described a dual role as external supervisors who visited peripheral facilities to carry out supervisory processes, but who also performed direct supervision within their own departments or wards.

“I do quarterly supervision in the health centres and at district I do go maybe twice a week to the wards just to see how the nurses are performing, and for the health centres I normally have a checklist which I use which has all components: maternity, infection prevention whatever…so I do use a checklist to do my supervision…and wherever I find the gaps I do on the job training.” (DNO, 172)

At facility level the departmental in-charges were expected to carry out immediate supervision of health workers.

Respondents described a variety of feedback mechanisms. A number of respondents were quick to stress that verbal feedback should be immediate and followed up with a written report. This verbal feedback could be given on an individual basis, or be presented to all facility staff at the end of a supervisory visit. Subsequent written reports were provided on a quarterly basis. One respondent described the use of action points for the next 3 months.

“Then when we come back from the supervision there’s also written feedback on what was discussed during the verbal feedback, so that in the next visit that we go to that facility we should also reflect on the action points that were documented…to see which have been done and which haven’t been done and what are the challenges.” (Deputy DHO, 261)

All managers at the district level were supported and supervised by zonal-level supervisors. “They do come now and again to see to it that actually we are administering our human resources properly. They have their own checklist which they bring when they come…a way of supervising as to what we are doing.” (Human Resources Manager, 161) Respondents valued this zonal oversight, as it encouraged them to focus on the HRM component of their work and provided an opportunity to problem solve and share good practice.

#### Tanzania

CHMT members reported high levels of responsibility for supervision of facilities in the district. Many teams aimed to visit health facilities every month, although the minimum requirement was that these visits should happen once a quarter. However, there was considerable variability in the frequency with which some facilities were supervised. Some CHMT members reported that they prioritised facilities from which they received complaints, where they then used “…another style of supervision, we do call it prompt supervision and we do this especially on places where we do receive complaints.” (DHS, 361)

Supervision was usually done as a team, with members of different departments going out to facilities together on scheduled visits. Some facilities were warned in advance that the teams were coming. Most respondents said they endeavoured to use supportive supervision and the techniques that this involved. Supervision guidelines, authorised by the Ministry of Health and Social Welfare, were used to carry out inspection of facilities. A supervision matrix and checklist were provided by the Department of Health in the District. These were based on national guidelines and focused on areas such as maternal and child health, immunisation, and voluntary counselling and testing for HIV/AIDS, but there were concerns that the checklists were not comprehensive enough to cover all necessary aspects, or lacked sufficient space to adequately capture all the issues. Another layer of record keeping involved completion of the MTUHA logbooks, which stipulated the criteria used to assess facility level performance. Participants agreed that these should be completed at each visit and remain in the health facility to leave a written record of the visit, allowing subsequent supervisors to follow up on outstanding action or issues. Many respondents felt that these provided a structure and target for the visit, as well as clear expectations and records of feedback.

CHMT supervisors also noted that spending time with health workers was an important component of supportive supervision. Some did this on an individual basis while others interacted with groups of health workers at the facility. They described observing daily activities and watching staff techniques, then following up with a discussion of strengths and weaknesses and plans made for improvement. There was widespread agreement that feedback should be given as soon as possible and that staff should feel supported and able to ask for help if needed. One mechanism cited was the use of the facility’s regular morning meetings as a platform to report on issues that had been resolved, or to discuss outstanding concerns and possible solutions. A written report was subsequently generated and sent back to the health facility, while other reports were filed in the CHMT offices.

### Assessing performance

There was a significant distinction between measuring facility level service provision and assessing individual staff performance. Participants in both countries were more likely to discuss indicators such as availability of supplies, number of deliveries and maternal mortality figures, as well as properly filled in registers and cleanliness of wards. This is unsurprising given their primary role of facility level oversight. However, there were cross-country differences in their discussion of the mechanisms available to C/DHMT staff to monitor the performance of health workers.

#### Malawi

There was a clear expectation that departmental in-charges would report to the DHMT on the performance of facility staff. However, few respondents discussed a mechanism to assess staff performance, or any system to oversee proper implementation of an assessment process.

“…there is gap in assessing supervision as well as assessing performance of staff…we also do use like the indicators that we have at the district to look at performance of the service, but not necessarily performance of the staff. If it’s performance of staff, it would be general in the sense that you would know that in reproductive health we are performing poorly because our indicators are poor, not looking at an individual performance.” (DNO, 122)

Even where DHMT members mentioned assessing performance themselves, there were inconsistencies in their reports of the criteria used. Any individual measures that were mentioned, such as punctuality, response time for on-call staff, absenteeism or staff reporting to work at recommended times, were notable in that they were not competency-based. Attempts to assess individual performance were complicated by lack of explicit expectations. Health workers were assumed to know the performance and quality expected of them based on their knowledge from school or in-service training. Many staff and facilities were reported to lack written job descriptions and, even if these were present, they tended to be generic and did not necessarily relate to the increased scope of practice of some cadres or changes to protocols for care. In these cases the DHMT relied on staff being familiar with the charts, procedure manuals and protocols that were supposed to be displayed in the facility to guide their performance. Staff meetings and departmental monthly meetings were expected to be used to inform health workers.

#### Tanzania

As in Malawi, some CHMT personnel relied on departmental supervisors to report to them on individual health worker performance, but many checked this for themselves as described above. Over half of the districts sampled in Tanzania explicitly discussed the use of a newly introduced mechanism, OPRAS, to define expectations and assess performance. Most were very positive, saying it provided a fair, open assessment from the health worker and the supervisor, with set targets and indicators that allowed progress to be verified and which made staff feel responsible. “Now that is the advantage with OPRAS. It defines clearly what a person has to deliver and in what quality. We agree upon this, everybody knows what is expected from him/her what she/he has to achieve this year, this month, semi-annually.” (DMO, 151) However, some participants were concerned that health workers at lower levels of the health service would find it difficult to articulate and quantify their performance aims and targets. In addition, although job descriptions were provided, the actual tasks staff did were not necessarily reflected in these documents. “They each have their own job description which is permanent but in practice it changes according to the environment.” (DHS, 522) Much of this was driven by circumstances. “They can do tasks which are not in the job description due to a shortage of employees. Yes, it is there, you find a medical attendant who has all the responsibilities which normally a doctor would do.” (DHS, 251)

### Challenges to implementation

Respondents in both countries described similar challenges that impacted on the frequency of supervisory visits and on C/DHMT autonomy. District management teams were involved in many other programmes, leading to conflicting responsibilities and multiple demands on their time, which were often given precedence over supervisory tasks. This caused particular difficulties where schedules for the whole team needed to be coordinated to ensure their availability. Financial constraints also caused frustration and led to cancellation or rescheduling of planned visits. “…we have supervisory systems and we aim to go there each month but we are stuck due to shortage of fuel and sometimes the delay of money reaching our account…The autonomy we have is hampered by lack of money, so what do you do?” (DHS, 402) This could lead to some remote facilities only being visited once a year. Given that external supervisory visits were sometimes their only link to more experienced health professionals, this could leave staff in rural facilities feeling abandoned and isolated.

Staff shortages, both of C/DHMT members themselves and staff at facility level, were described as a major impediment to effective supervision. Within facilities, absolute shortages of staff were also cited as a challenge to adequate supervision, particularly the dearth of the more senior grades, such as doctors and registered nurse-midwives, who were expected to supervise facility staff. In addition, when district managers visited facilities the shortage of lower level cadres hampered effective supervision.

“The workload is such…and there is such a shortage of staff that sometimes instead of going for supervision you have to assist the person you were going to supervise because they are so overburdened with work. You work, so in most cases even the supervision becomes minimal because you have to join them in dealing with patients, rather than sitting and supervising or observing.” (Nursing Officer, 461)

## Discussion

This research revealed divergent attitudes regarding the nature and purpose of the supervision process in the two countries studied. These attitudes are nested in the policy environment and the value or support that is given to the supervision function and can have a significant impact on the implementation of supervision activities. In Tanzania, where there is policy-level attention to the importance of supportive supervision as a tool for advancing health sector objectives, CHMT attitudes clearly suggested a paradigm of teaching, problem solving and improvement. This reflects a national commitment, reinforced with clear mechanisms, structures and shared expectations, that views supportive supervision and the attitudes upon which this is based as a necessary part of the HRM process. However, in Malawi, where DHMT members described a context that lacked a systematic, accountable supervision structure, with unclear criteria and assumed expectations of staff performance, supervision practice was dependent on the attitudes and priorities of supervisors. The prevailing supervision paradigm has important repercussions for health worker motivation, retention and performance. Fault-finding inspection models coupled with a lack of transparency in HRM processes and criteria can have negative impacts on staff motivation [4,22]. Conversely, supportive supervision practices can influence a range of outcomes, including job satisfaction [39], turnover intention [40] and performance [41].

Central to the discussion about integrated supervision at the district level is the need for clarity and support for the DHMT in their role. Participants in this study revealed complex demands in their capacity as managerial supervisors carrying out external supervision to lower-level health facilities, combined with clinical supervisory responsibility either within the district hospital in which they were based, or driven by staff shortages or lack of senior cadres in smaller district facilities. This demonstrates the all-encompassing conception of ‘supervision’ in these contexts and adds to the lack of a common understanding of supervision’s purpose and role within the HRM function. It is clear that the DHMT need to monitor and evaluate supervision processes within the district, but they do not have the time or resources to supervise individual staff. Their effort would be most effectively targeted at setting up and monitoring the mechanisms at facility level that support staff performance, rather than overseeing individual health workers. CHMT personnel in Tanzania had the new OPRAS system in place that should address some of these issues. In Malawi, however, respondents voiced concerns about the lack of mechanisms to define and assess individual performance, outlining a clear discrepancy between their recognition that health workers need to be supported and appreciated for the work they do and the lack of mechanisms to measure or reward this effort. The implications of this for health worker motivation and retention have been documented elsewhere [2-5,11,22,23]. Even where individual level performance indicators were cited, they were not competency-based. This is of concern in the context of scaling up health worker numbers and the changes to scope of practice that have been introduced to increase access to basic emergency obstetric care. The influx of large numbers of newly qualified staff, who may lack the skills and experience to perform well, coupled with the absence of an effective supervision system, has obvious ramifications for quality of care [42] and is increasingly recognised by managers as an area to be addressed. Enhanced mechanisms at district level, such as audit and feedback to reduce maternal complications [43], could justifiably fall within the DHMT’s supervision remit and form part of a suite of measures to support performance and accountability.

None of these measures can be implemented without sufficient senior staff with the requisite knowledge and skills. These supervision capacity constraints, particularly in more rural areas, will need to be addressed in order to create the sort of supportive workplace environment that will attract and retain health workers [44]. Even when supervisory staff are available, there are challenges to carrying out scheduled supervision visits. Visits are often postponed due to over commitment with other, perceived higher priority HRM roles, inadequate finances, or transport and accessibility problems, underlining the need for proper prioritisation and adequate resourcing of supervision as a key HRM activity. This study reported infrequent supervision of remote facilities, which may contribute to absenteeism and reduced performance [45].

Supervision ought to be a formalised HRM tool, which is integrated into the day-to-day functioning of a health sector organisation and in which supervisors encourage quality improvement and genuinely value their staff [1]. It should take into account health workers’ personal goals and needs, while working to support good practice and to correct shortcomings [11]. Supervisors themselves need to have good leadership skills and treat all employees fairly [10]. The concept of supportive supervision focuses particularly on the importance of mentoring, joint problem solving and two-way communication. It emphasises that supervisors must have the solid technical knowledge and skills needed to perform tasks, the know-how to access additional support as needed, and have time to meet with the staff they supervise [46].

Without functional and supportive supervision, it is unlikely that incentive systems aiming to retain health workers will be effective [47]. With it, health workers are more likely to experience a sense of self-efficacy and feel motivated and satisfied [11]. A focus on supportive supervision engenders a mind-set where teams of health workers identify their own challenges and achieve results with support from their supervisors. It moves away from an ‘inspection and blame’ model to one characterised by ‘support, shared responsibility and problem solving’. This can address motivators such as achievement (goals are clear and achievable), recognition (performance is recognised), responsibility (health workers feel ownership of their work) and advancement (performance and commitment are rewarded) [10]. Ultimately, the implementation of supervision systems at the national level requires commitment and support from leadership to promote supervision and remove impediments to its implementation [48]. The intervention of governments and their partners is crucial in translating the language and policy of supervision into improvements in the motivation, satisfaction and retention of health workers.

## Conclusion

HRM aims to enable motivated, competent staff to meet health sector objectives. Supervision is one mechanism that helps to achieve this and is particularly important when staff operate in a challenging work environment or in the context of task shifting. In order to understand the gaps between practice and policy it is important to include the perspectives of those staff tasked with carrying out the supervisory role. The findings of this study have important implications for policy makers. National supervision plans are only as good as the supervisors who implement them and can fail if the underlying ethos and attitude towards supervision is not clear to all health workers involved in the supervision process. This study revealed divergent attitudes to supervision and differing perceptions of the level of support for this crucial aspect of HRM, particularly in Malawi. Key to the provision of supportive supervision is the presence of an effective HRM structure and practice, at both national and district levels, which is appropriately prioritised. Policy level attention and commitment is crucial to ensure an adequately resourced, systematic, structured process at district level that is based on common understandings of the role and purpose of supervision.

### Limitations

Data for this element of the HSSE study were drawn from a purposive sample of C/DHMT members, where at least two of the three key cadres identified were available during the time when data collection teams were present in their district. In addition, the logistics of the data collection process meant that a target was set in advance for the number of interviews that could be collected. This may have led to some bias, as districts where at least two of these senior staff were available may not be representative of the entire C/DHMT population.

## Endnotes

^a^Basic EmOC is comprised of seven signal functions: 1. Administer parenteral antibiotics; 2. Administer uterotonic drugs; 3. Administer parenteral anticonvulsants for pre-eclampsia and eclampsia; 4. Manual removal of placenta; 5. Removal of retained products of conception; 6. Assisted vaginal delivery; 7. Neonatal resuscitation. An additional two signal functions indicate comprehensive EmOC: 8. Perform emergency obstetric surgery (e.g. caesarean section); 9. Perform blood transfusion.

## Abbreviations

CHMT: Council Health Management Teams; DHMT: District Health Management Teams; DHO: District Health Officer; DHS: District Health Secretary; DMO: District Medical Officer; DNO: District Nursing Officer; EHRP: Emergency Human Resources Programme; EmOC: Emergency obstetric care; HRM: Human resource management; HSSE: Health Systems Strengthening for Equity: The Power and Potential of Mid-Level Providers; OPRAS: Open Performance Review and Appraisal System; RCH: Reproductive and Child Health.

## Competing interests

The authors declare that they have no competing interests.

## Authors’ contributions

SB participated in the study design, data collection/analysis and drafted this paper. EM participated in the study design and data analysis and contributed to the paper. FK participated in the study design, data collection and data analysis (particularly in Malawi). HM participated in the study design, data collection and data analysis (particularly in Tanzania). HdP managed the project and participated in the study design, data collection and data analysis, and contributed to the paper. RW participated in the study design, data collection/analysis and contributed to the paper. CB contributed to the literature review for the paper. All authors read and approved the final manuscript.
